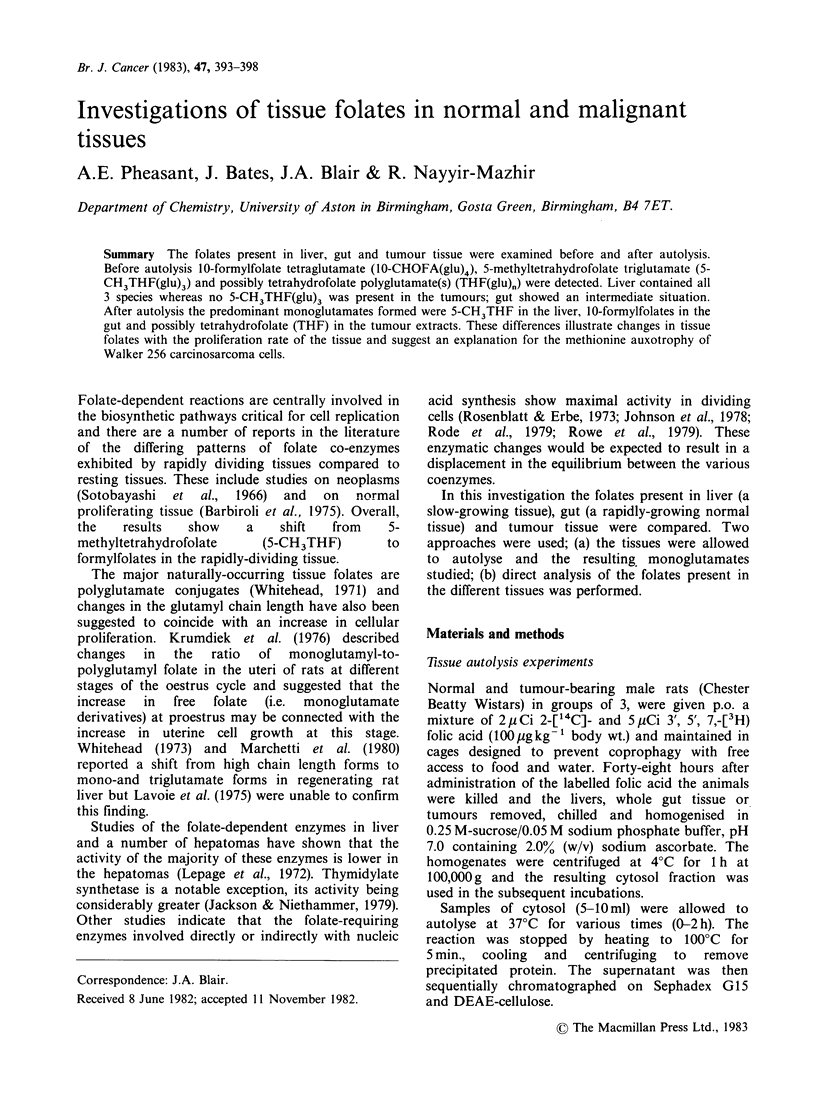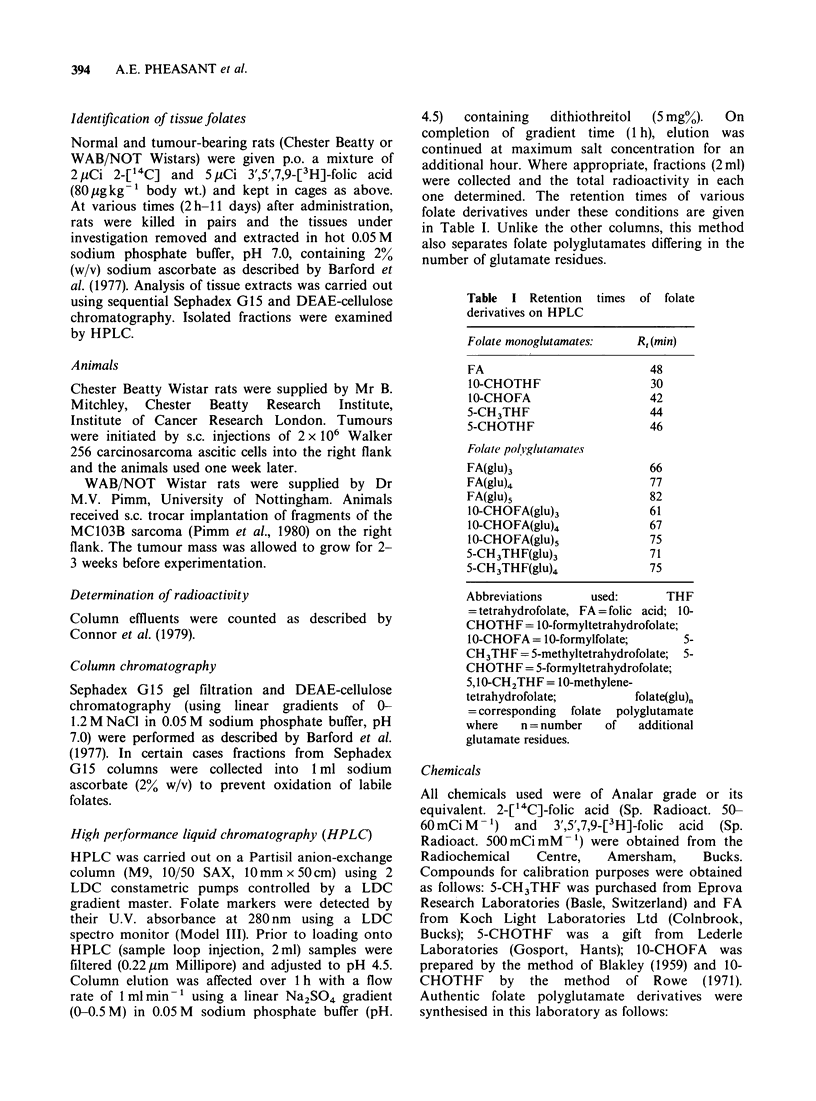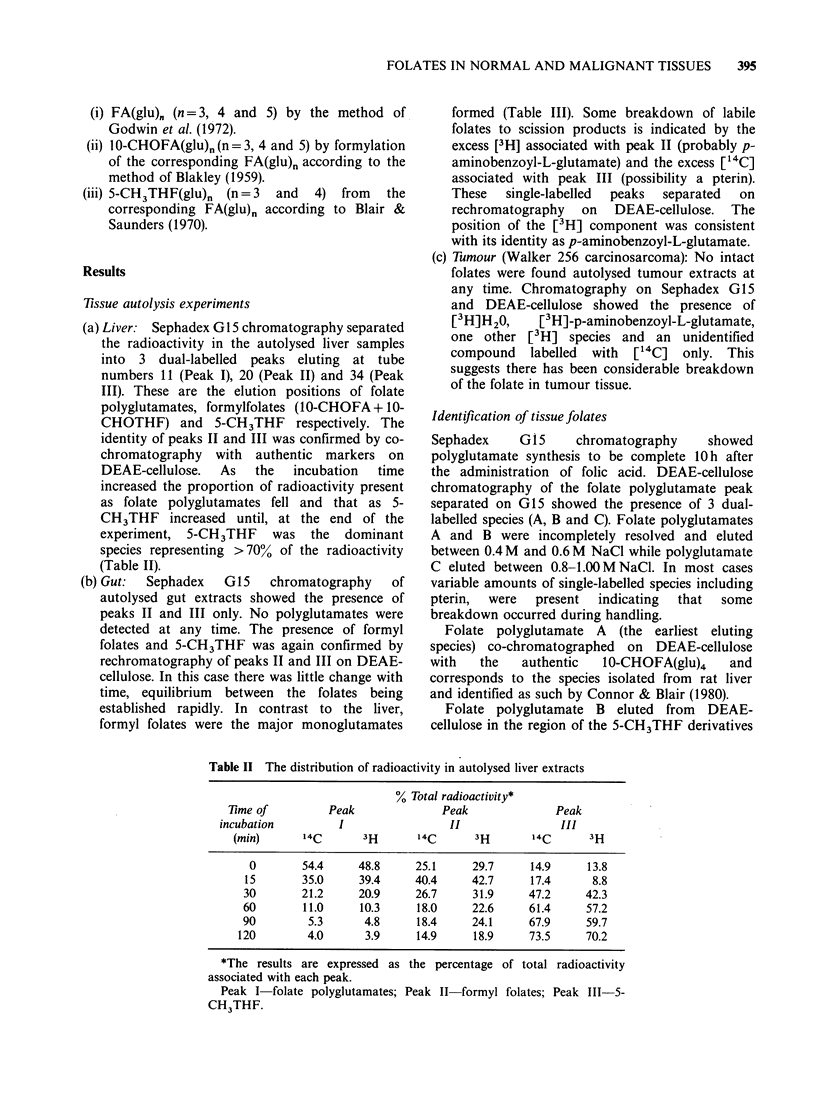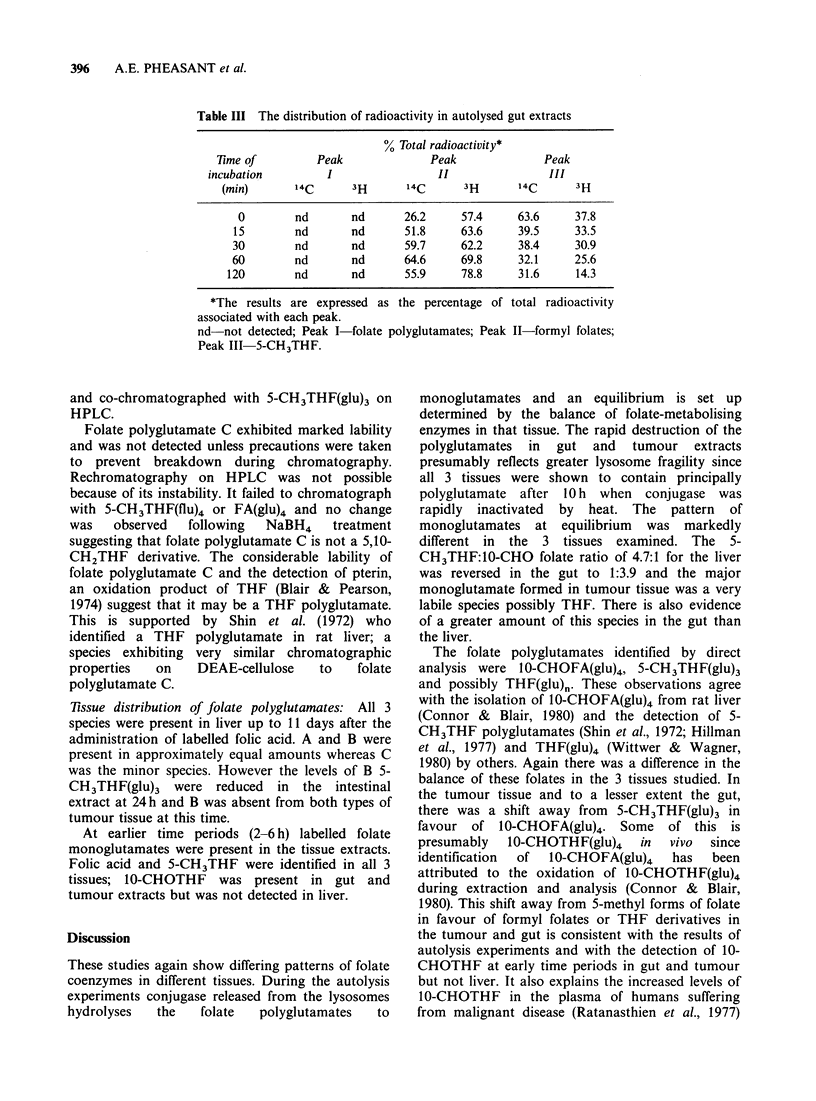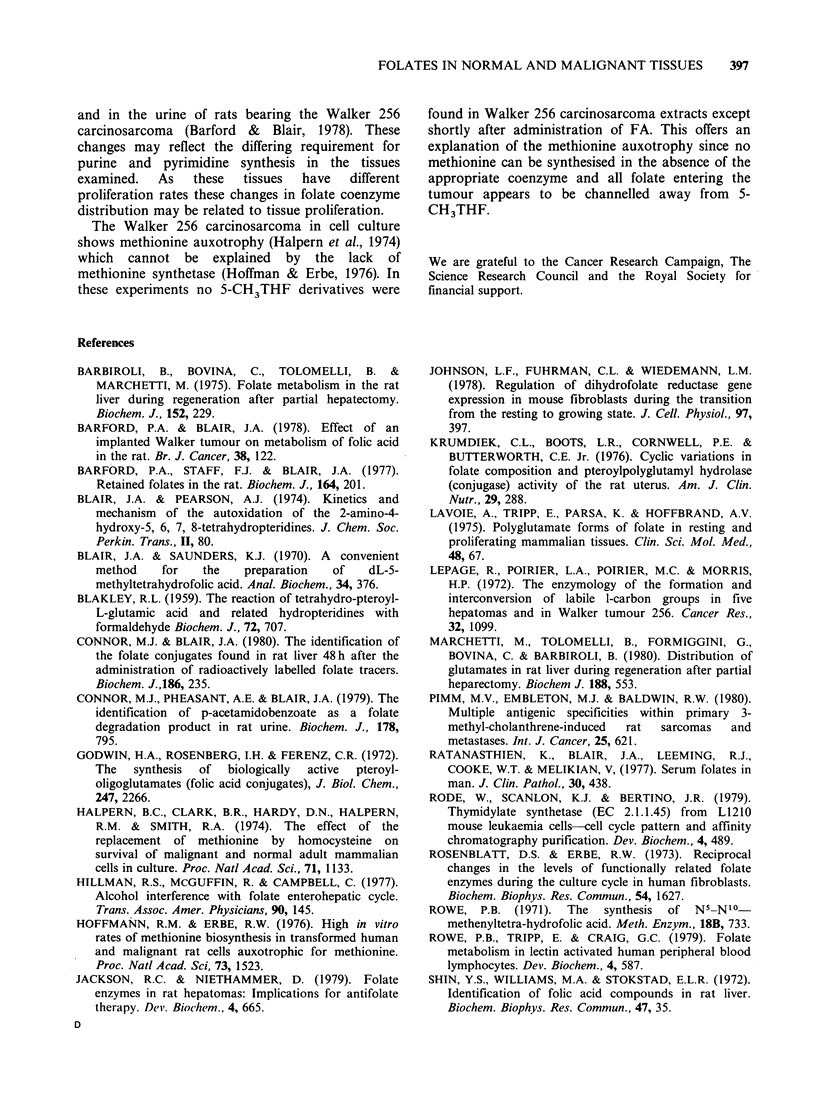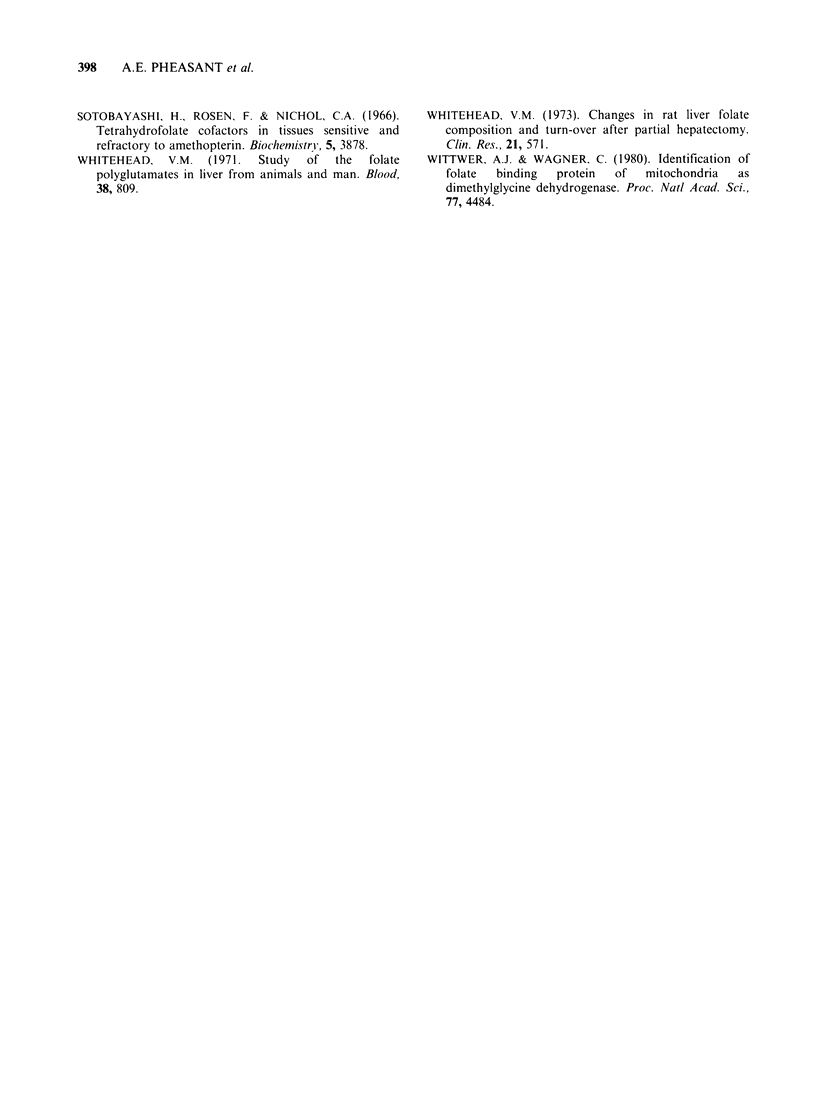# Investigations of tissue folates in normal and malignant tissues.

**DOI:** 10.1038/bjc.1983.59

**Published:** 1983-03

**Authors:** A. E. Pheasant, J. Bates, J. A. Blair, R. Nayyir-Mazhir

## Abstract

The folates present in liver, gut and tumour tissue were examined before and after autolysis. Before autolysis 10-formylfolate tetraglutamate (10-CHOFA(glu)4), 5-methyltetrahydrofolate triglutamate (5-CH3THF(glu)3) and possibly tetrahydrofolate polyglutamate(s) (THF(glu)n) were detected. Liver contained all 3 species whereas no 5-CH3THF(glu)3 was present in the tumours; gut showed an intermediate situation. After autolysis the predominant monoglutamates formed were 5-CH3THF in the liver, 10-formylfolates in the gut and possibly tetrahydrofolate (THF) in the tumour extracts. These differences illustrate changes in tissue folates with the proliferation rate of the tissue and suggest an explanation for the methionine auxotrophy of Walker 256 carcinosarcoma cells.


*Br. J. Cancer* (1983), **47**, 393–398

### Investigations of tissue folates in normal and malignant tissues

A.E. Pheasant, J. Bates, J.A. Blair & R. Nayyir-Mazhir

*Department of Chemistry, University of Aston in Birmingham, Gosta Green, Birmingham, B4 7ET.*

**Summary** The folates present in liver, gut and tumour tissue were examined before and after autolysis. Before autolysis 10-formylfolate tetraglutamate (10-CHOFA$(glu)_4$), 5-methyltetrahydrofolate triglutamate (5-$CH_3$THF$(glu)_3$) and possibly tetrahydrofolate polyglutamate(s) (THF$(glu)_n$) were detected. Liver contained all 3 species whereas no 5-$CH_3$THF$(glu)_3$ was present in the tumours; gut showed an intermediate situation. After autolysis the predominant monoglutamates formed were 5-$CH_3$THF in the liver, 10-formylfolates in the gut and possibly tetrahydrofolate (THF) in the tumour extracts. These differences illustrate changes in tissue folates with the proliferation rate of the tissue and suggest an explanation for the methionine auxotrophy of Walker 256 carcinosarcoma cells.

Folate-dependent reactions are centrally involved in the biosynthetic pathways critical for cell replication and there are a number of reports in the literature of the differing patterns of folate co-enzymes exhibited by rapidly dividing tissues compared to resting tissues. These include studies on neoplasms (Sotobayashi *et al.*, 1966) and on normal proliferating tissue (Barbiroli *et al.*, 1975). Overall, the results show a shift from 5-methyltetrahydrofolate (5-$CH_3$THF) to formylfolates in the rapidly-dividing tissue.

The major naturally-occurring tissue folates are polyglutamate conjugates (Whitehead, 1971) and changes in the glutamyl chain length have also been suggested to coincide with an increase in cellular proliferation. Krumdiek *et al.* (1976) described changes in the ratio of monoglutamyl-to-polyglutamyl folate in the uteri of rats at different stages of the oestrus cycle and suggested that the increase in free folate (i.e. monoglutamate derivatives) at proestrus may be connected with the increase in uterine cell growth at this stage. Whitehead (1973) and Marchetti *et al.* (1980) reported a shift from high chain length forms to mono-and triglutamate forms in regenerating rat liver but Lavoie *et al.* (1975) were unable to confirm this finding.

Studies of the folate-dependent enzymes in liver and a number of hepatomas have shown that the activity of the majority of these enzymes is lower in the hepatomas (Lepage *et al.*, 1972). Thymidylate synthetase is a notable exception, its activity being considerably greater (Jackson & Niethammer, 1979). Other studies indicate that the folate-requiring enzymes involved directly or indirectly with nucleic acid synthesis show maximal activity in dividing cells (Rosenblatt & Erbe, 1973; Johnson *et al.*, 1978; Rode *et al.*, 1979; Rowe *et al.*, 1979). These enzymatic changes would be expected to result in a displacement in the equilibrium between the various coenzymes.

In this investigation the folates present in liver (a slow-growing tissue), gut (a rapidly-growing normal tissue) and tumour tissue were compared. Two approaches were used; (a) the tissues were allowed to autolyse and the resulting monoglutamates studied; (b) direct analysis of the folates present in the different tissues was performed.

### Materials and methods

### *Tissue autolysis experiments*

Normal and tumour-bearing male rats (Chester Beatty Wistars) in groups of 3, were given p.o. a mixture of 2 $\mu$Ci 2-[$^{14}$C]- and 5 $\mu$Ci 3′, 5′, 7,-[$^{3}$H) folic acid (100 $\mu$g kg$^{-1}$ body wt.) and maintained in cages designed to prevent coprophagy with free access to food and water. Forty-eight hours after administration of the labelled folic acid the animals were killed and the livers, whole gut tissue or tumours removed, chilled and homogenised in 0.25 M-sucrose/0.05 M sodium phosphate buffer, pH 7.0 containing 2.0% (w/v) sodium ascorbate. The homogenates were centrifuged at 4°C for 1 h at 100,000 g and the resulting cytosol fraction was used in the subsequent incubations.

Samples of cytosol (5–10 ml) were allowed to autolyse at 37°C for various times (0–2 h). The reaction was stopped by heating to 100°C for 5 min., cooling and centrifuging to remove precipitated protein. The supernatant was then sequentially chromatographed on Sephadex G15 and DEAE-cellulose.

Correspondence: J.A. Blair.

Received 8 June 1982; accepted 11 November 1982.

© The Macmillan Press Ltd., 1983

*Identification of tissue folates*

Normal and tumour-bearing rats (Chester Beatty or WAB/NOT Wistars) were given p.o. a mixture of 2 μCi 2-[$^{14}C$] and 5 μCi 3′,5′,7,9-[$^{3}H$]-folic acid (80 μg kg$^{-1}$ body wt.) and kept in cages as above. At various times (2 h–11 days) after administration, rats were killed in pairs and the tissues under investigation removed and extracted in hot 0.05 M sodium phosphate buffer, pH 7.0, containing 2% (w/v) sodium ascorbate as described by Barford *et al.* (1977). Analysis of tissue extracts was carried out using sequential Sephadex G15 and DEAE-cellulose chromatography. Isolated fractions were examined by HPLC.

*Animals*

Chester Beatty Wistar rats were supplied by Mr B. Mitchley, Chester Beatty Research Institute, Institute of Cancer Research London. Tumours were initiated by s.c. injections of $2 \times 10^6$ Walker 256 carcinosarcoma ascitic cells into the right flank and the animals used one week later.

WAB/NOT Wistar rats were supplied by Dr M.V. Pimm, University of Nottingham. Animals received s.c. trocar implantation of fragments of the MC103B sarcoma (Pimm *et al.*, 1980) on the right flank. The tumour mass was allowed to grow for 2–3 weeks before experimentation.

*Determination of radioactivity*

Column effluents were counted as described by Connor *et al.* (1979).

*Column chromatography*

Sephadex G15 gel filtration and DEAE-cellulose chromatography (using linear gradients of 0–1.2 M NaCl in 0.05 M sodium phosphate buffer, pH 7.0) were performed as described by Barford *et al.* (1977). In certain cases fractions from Sephadex G15 columns were collected into 1 ml sodium ascorbate (2% w/v) to prevent oxidation of labile folates.

*High performance liquid chromatography (HPLC)*

HPLC was carried out on a Partisil anion-exchange column (M9, 10/50 SAX, 10 mm × 50 cm) using 2 LDC constametric pumps controlled by a LDC gradient master. Folate markers were detected by their U.V. absorbance at 280 nm using a LDC spectro monitor (Model III). Prior to loading onto HPLC (sample loop injection, 2 ml) samples were filtered (0.22 μm Millipore) and adjusted to pH 4.5. Column elution was affected over 1 h with a flow rate of 1 ml min$^{-1}$ using a linear $Na_2SO_4$ gradient (0–0.5 M) in 0.05 M sodium phosphate buffer (pH. 4.5) containing dithiothreitol (5 mg%). On completion of gradient time (1 h), elution was continued at maximum salt concentration for an additional hour. Where appropriate, fractions (2 ml) were collected and the total radioactivity in each one determined. The retention times of various folate derivatives under these conditions are given in Table I. Unlike the other columns, this method also separates folate polyglutamates differing in the number of glutamate residues.

**Table I** Retention times of folate derivatives on HPLC

| *Folate monoglutamates:* | $R_t$ *(min)* |
|---|---|
| FA | 48 |
| 10-CHOTHF | 30 |
| 10-CHOFA | 42 |
| 5-$CH_3$THF | 44 |
| 5-CHOTHF | 46 |
| *Folate polyglutamates* | |
| FA(glu)$_3$ | 66 |
| FA(glu)$_4$ | 77 |
| FA(glu)$_5$ | 82 |
| 10-CHOFA(glu)$_3$ | 61 |
| 10-CHOFA(glu)$_4$ | 67 |
| 10-CHOFA(glu)$_5$ | 75 |
| 5-$CH_3$THF(glu)$_3$ | 71 |
| 5-$CH_3$THF(glu)$_4$ | 75 |

Abbreviations used: THF = tetrahydrofolate, FA = folic acid; 10-CHOTHF = 10-formyltetrahydrofolate; 10-CHOFA = 10-formylfolate; 5-$CH_3$THF = 5-methyltetrahydrofolate; 5-CHOTHF = 5-formyltetrahydrofolate; 5,10-$CH_2$THF = 10-methylene-tetrahydrofolate; folate(glu)$_n$ = corresponding folate polyglutamate where n = number of additional glutamate residues.

*Chemicals*

All chemicals used were of Analar grade or its equivalent. 2-[$^{14}C$]-folic acid (Sp. Radioact. 50–60 mCi M$^{-1}$) and 3′,5′,7,9-[$^{3}H$]-folic acid (Sp. Radioact. 500 mCi mM$^{-1}$) were obtained from the Radiochemical Centre, Amersham, Bucks. Compounds for calibration purposes were obtained as follows: 5-$CH_3$THF was purchased from Eprova Research Laboratories (Basle, Switzerland) and FA from Koch Light Laboratories Ltd (Colnbrook, Bucks); 5-CHOTHF was a gift from Lederle Laboratories (Gosport, Hants); 10-CHOFA was prepared by the method of Blakley (1959) and 10-CHOTHF by the method of Rowe (1971). Authentic folate polyglutamate derivatives were synthesised in this laboratory as follows:

(i) FA(glu)$_n$ ($n=3$, 4 and 5) by the method of Godwin *et al.* (1972).

(ii) 10-CHOFA(glu)$_n$ (n = 3, 4 and 5) by formylation of the corresponding FA(glu)$_n$ according to the method of Blakley (1959).

(iii) 5-$CH_3$THF(glu)$_n$ (n = 3 and 4) from the corresponding FA(glu)$_n$ according to Blair & Saunders (1970).

### Results

### *Tissue autolysis experiments*

(a) *Liver:* Sephadex G15 chromatography separated the radioactivity in the autolysed liver samples into 3 dual-labelled peaks eluting at tube numbers 11 (Peak I), 20 (Peak II) and 34 (Peak III). These are the elution positions of folate polyglutamates, formylfolates (10-CHOFA + 10-CHOTHF) and 5-$CH_3$THF respectively. The identity of peaks II and III was confirmed by co-chromatography with authentic markers on DEAE-cellulose. As the incubation time increased the proportion of radioactivity present as folate polyglutamates fell and that as 5-$CH_3$THF increased until, at the end of the experiment, 5-$CH_3$THF was the dominant species representing >70% of the radioactivity (Table II).

(b) *Gut:* Sephadex G15 chromatography of autolysed gut extracts showed the presence of peaks II and III only. No polyglutamates were detected at any time. The presence of formyl folates and 5-$CH_3$THF was again confirmed by rechromatography of peaks II and III on DEAE-cellulose. In this case there was little change with time, equilibrium between the folates being established rapidly. In contrast to the liver, formyl folates were the major monoglutamates formed (Table III). Some breakdown of labile folates to scission products is indicated by the excess [$^3$H] associated with peak II (probably *p*-aminobenzoyl-L-glutamate) and the excess [$^{14}$C] associated with peak III (possibility a pterin). These single-labelled peaks separated on rechromatography on DEAE-cellulose. The position of the [$^3$H] component was consistent with its identity as *p*-aminobenzoyl-L-glutamate.

(c) *Tumour* (Walker 256 carcinosarcoma): No intact folates were found autolysed tumour extracts at any time. Chromatography on Sephadex G15 and DEAE-cellulose showed the presence of [$^3$H]$H_2$0, [$^3$H]-p-aminobenzoyl-L-glutamate, one other [$^3$H] species and an unidentified compound labelled with [$^{14}$C] only. This suggests there has been considerable breakdown of the folate in tumour tissue.

### *Identification of tissue folates*

Sephadex G15 chromatography showed polyglutamate synthesis to be complete 10 h after the administration of folic acid. DEAE-cellulose chromatography of the folate polyglutamate peak separated on G15 showed the presence of 3 dual-labelled species (A, B and C). Folate polyglutamates A and B were incompletely resolved and eluted between 0.4 M and 0.6 M NaCl while polyglutamate C eluted between 0.8–1.00 M NaCl. In most cases variable amounts of single-labelled species including pterin, were present indicating that some breakdown occurred during handling.

Folate polyglutamate A (the earliest eluting species) co-chromatographed on DEAE-cellulose with the authentic 10-CHOFA(glu)$_4$ and corresponds to the species isolated from rat liver and identified as such by Connor & Blair (1980).

Folate polyglutamate B eluted from DEAE-cellulose in the region of the 5-$CH_3$THF derivatives

**Table II** The distribution of radioactivity in autolysed liver extracts

| *Time of incubation (min)* | *% Total radioactivity** *Peak I* $^{14}$C | *Peak I* $^{3}$H | *Peak II* $^{14}$C | *Peak II* $^{3}$H | *Peak III* $^{14}$C | *Peak III* $^{3}$H |
|---|---|---|---|---|---|---|
| 0 | 54.4 | 48.8 | 25.1 | 29.7 | 14.9 | 13.8 |
| 15 | 35.0 | 39.4 | 40.4 | 42.7 | 17.4 | 8.8 |
| 30 | 21.2 | 20.9 | 26.7 | 31.9 | 47.2 | 42.3 |
| 60 | 11.0 | 10.3 | 18.0 | 22.6 | 61.4 | 57.2 |
| 90 | 5.3 | 4.8 | 18.4 | 24.1 | 67.9 | 59.7 |
| 120 | 4.0 | 3.9 | 14.9 | 18.9 | 73.5 | 70.2 |

*The results are expressed as the percentage of total radioactivity associated with each peak.

Peak I—folate polyglutamates; Peak II—formyl folates; Peak III—5-$CH_3$THF.

**Table III** The distribution of radioactivity in autolysed gut extracts

| *Time of incubation (min)* | *% Total radioactivity** *Peak I* $^{14}C$ | $^{3}H$ | *Peak II* $^{14}C$ | $^{3}H$ | *Peak III* $^{14}C$ | $^{3}H$ |
|---|---|---|---|---|---|---|
| 0 | nd | nd | 26.2 | 57.4 | 63.6 | 37.8 |
| 15 | nd | nd | 51.8 | 63.6 | 39.5 | 33.5 |
| 30 | nd | nd | 59.7 | 62.2 | 38.4 | 30.9 |
| 60 | nd | nd | 64.6 | 69.8 | 32.1 | 25.6 |
| 120 | nd | nd | 55.9 | 78.8 | 31.6 | 14.3 |

*The results are expressed as the percentage of total radioactivity associated with each peak.
nd—not detected; Peak I—folate polyglutamates; Peak II—formyl folates; Peak III—5-$CH_3$THF.

and co-chromatographed with 5-$CH_3$THF$(glu)_3$ on HPLC.

Folate polyglutamate C exhibited marked lability and was not detected unless precautions were taken to prevent breakdown during chromatography. Rechromatography on HPLC was not possible because of its instability. It failed to chromatograph with 5-$CH_3$THF$(flu)_4$ or FA$(glu)_4$ and no change was observed following $NaBH_4$ treatment suggesting that folate polyglutamate C is not a 5,10-$CH_2$THF derivative. The considerable lability of folate polyglutamate C and the detection of pterin, an oxidation product of THF (Blair & Pearson, 1974) suggest that it may be a THF polyglutamate. This is supported by Shin *et al.* (1972) who identified a THF polyglutamate in rat liver; a species exhibiting very similar chromatographic properties on DEAE-cellulose to folate polyglutamate C.

*Tissue distribution of folate polyglutamates:* All 3 species were present in liver up to 11 days after the administration of labelled folic acid. A and B were present in approximately equal amounts whereas C was the minor species. However the levels of B 5-$CH_3$THF$(glu)_3$ were reduced in the intestinal extract at 24 h and B was absent from both types of tumour tissue at this time.

At earlier time periods (2–6 h) labelled folate monoglutamates were present in the tissue extracts. Folic acid and 5-$CH_3$THF were identified in all 3 tissues; 10-CHOTHF was present in gut and tumour extracts but was not detected in liver.

### Discussion

These studies again show differing patterns of folate coenzymes in different tissues. During the autolysis experiments conjugase released from the lysosomes hydrolyses the folate polyglutamates to monoglutamates and an equilibrium is set up determined by the balance of folate-metabolising enzymes in that tissue. The rapid destruction of the polyglutamates in gut and tumour extracts presumably reflects greater lysosome fragility since all 3 tissues were shown to contain principally polyglutamate after 10 h when conjugase was rapidly inactivated by heat. The pattern of monoglutamates at equilibrium was markedly different in the 3 tissues examined. The 5-$CH_3$THF:10-CHO folate ratio of 4.7:1 for the liver was reversed in the gut to 1:3.9 and the major monoglutamate formed in tumour tissue was a very labile species possibly THF. There is also evidence of a greater amount of this species in the gut than the liver.

The folate polyglutamates identified by direct analysis were 10-CHOFA$(glu)_4$, 5-$CH_3$THF$(glu)_3$ and possibly THF$(glu)_n$. These observations agree with the isolation of 10-CHOFA$(glu)_4$ from rat liver (Connor & Blair, 1980) and the detection of 5-$CH_3$THF polyglutamates (Shin *et al.*, 1972; Hillman *et al.*, 1977) and THF$(glu)_4$ (Wittwer & Wagner, 1980) by others. Again there was a difference in the balance of these folates in the 3 tissues studied. In the tumour tissue and to a lesser extent the gut, there was a shift away from 5-$CH_3$THF$(glu)_3$ in favour of 10-CHOFA$(glu)_4$. Some of this is presumably 10-CHOTHF$(glu)_4$ *in vivo* since identification of 10-CHOFA$(glu)_4$ has been attributed to the oxidation of 10-CHOTHF$(glu)_4$ during extraction and analysis (Connor & Blair, 1980). This shift away from 5-methyl forms of folate in favour of formyl folates or THF derivatives in the tumour and gut is consistent with the results of autolysis experiments and with the detection of 10-CHOTHF at early time periods in gut and tumour but not liver. It also explains the increased levels of 10-CHOTHF in the plasma of humans suffering from malignant disease (Ratanasthien *et al.*, 1977)

and in the urine of rats bearing the Walker 256 carcinosarcoma (Barford & Blair, 1978). These changes may reflect the differing requirement for purine and pyrimidine synthesis in the tissues examined. As these tissues have different proliferation rates these changes in folate coenzyme distribution may be related to tissue proliferation.

The Walker 256 carcinosarcoma in cell culture shows methionine auxotrophy (Halpern *et al.*, 1974) which cannot be explained by the lack of methionine synthetase (Hoffman & Erbe, 1976). In these experiments no 5-$CH_3$THF derivatives were found in Walker 256 carcinosarcoma extracts except shortly after administration of FA. This offers an explanation of the methionine auxotrophy since no methionine can be synthesised in the absence of the appropriate coenzyme and all folate entering the tumour appears to be channelled away from 5-$CH_3$THF.

We are grateful to the Cancer Research Campaign, The Science Research Council and the Royal Society for financial support.

**References**

BARBIROLI, B., BOVINA, C., TOLOMELLI, B. & MARCHETTI, M. (1975). Folate metabolism in the rat liver during regeneration after partial hepatectomy. *Biochem. J.*, **152,** 229.

BARFORD, P.A. & BLAIR, J.A. (1978). Effect of an implanted Walker tumour on metabolism of folic acid in the rat. *Br. J. Cancer,* **38,** 122.

BARFORD, P.A., STAFF, F.J. & BLAIR, J.A. (1977). Retained folates in the rat. *Biochem. J.*, **164,** 201.

BLAIR, J.A. & PEARSON, A.J. (1974). Kinetics and mechanism of the autoxidation of the 2-amino-4-hydroxy-5, 6, 7, 8-tetrahydropteridines. *J. Chem. Soc. Perkin. Trans.*, **II,** 80.

BLAIR, J.A. & SAUNDERS, K.J. (1970). A convenient method for the preparation of dL-5-methyltetrahydrofolic acid. *Anal. Biochem.*, **34,** 376.

BLAKLEY, R.L. (1959). The reaction of tetrahydro-pteroyl-L-glutamic acid and related hydropteridines with formaldehyde *Biochem. J.*, **72,** 707.

CONNOR, M.J. & BLAIR, J.A. (1980). The identification of the folate conjugates found in rat liver 48 h after the administration of radioactively labelled folate tracers. *Biochem. J.*,**186,** 235.

CONNOR, M.J., PHEASANT, A.E. & BLAIR, J.A. (1979). The identification of p-acetamidobenzoate as a folate degradation product in rat urine. *Biochem. J.*, **178,** 795.

GODWIN, H.A., ROSENBERG, I.H. & FERENZ, C.R. (1972). The synthesis of biologically active pteroyl-oligoglutamates (folic acid conjugates), *J. Biol. Chem.*, **247,** 2266.

HALPERN, B.C., CLARK, B.R., HARDY, D.N., HALPERN, R.M. & SMITH, R.A. (1974). The effect of the replacement of methionine by homocysteine on survival of malignant and normal adult mammalian cells in culture. *Proc. Natl Acad. Sci.*, **71,** 1133.

HILLMAN, R.S., MCGUFFIN, R. & CAMPBELL, C. (1977). Alcohol interference with folate enterohepatic cycle. *Trans. Assoc. Amer. Physicians,* **90,** 145.

HOFFMANN, R.M. & ERBE, R.W. (1976). High *in vitro* rates of methionine biosynthesis in transformed human and malignant rat cells auxotrophic for methionine. *Proc. Natl Acad. Sci,* **73,** 1523.

JACKSON, R.C. & NIETHAMMER, D. (1979). Folate enzymes in rat hepatomas: Implications for antifolate therapy. *Dev. Biochem.*, **4,** 665.

JOHNSON, L.F., FUHRMAN, C.L. & WIEDEMANN, L.M. (1978). Regulation of dihydrofolate reductase gene expression in mouse fibroblasts during the transition from the resting to growing state. *J. Cell. Physiol.*, **97,** 397.

KRUMDIEK, C.L., BOOTS, L.R., CORNWELL, P.E. & BUTTERWORTH, C.E. Jr. (1976). Cyclic variations in folate composition and pteroylpolyglutamyl hydrolase (conjugase) activity of the rat uterus. *Am. J. Clin. Nutr.*, **29,** 288.

LAVOIE, A., TRIPP, E., PARSA, K. & HOFFBRAND, A.V. (1975). Polyglutamate forms of folate in resting and proliferating mammalian tissues. *Clin. Sci. Mol. Med.*, **48,** 67.

LEPAGE, R., POIRIER, L.A., POIRIER, M.C. & MORRIS, H.P. (1972). The enzymology of the formation and interconversion of labile l-carbon groups in five hepatomas and in Walker tumour 256. *Cancer Res.*, **32,** 1099.

MARCHETTI, M., TOLOMELLI, B., FORMIGGINI, G., BOVINA, C. & BARBIROLI, B. (1980). Distribution of glutamates in rat liver during regeneration after partial heparectomy. *Biochem J.* **188,** 553.

PIMM, M.V., EMBLETON, M.J. & BALDWIN, R.W. (1980). Multiple antigenic specificities within primary 3-methyl-cholanthrene-induced rat sarcomas and metastases. *Int. J. Cancer,* **25,** 621.

RATANASTHIEN, K., BLAIR, J.A., LEEMING, R.J., COOKE, W.T. & MELIKIAN, V, (1977). Serum folates in man. *J. Clin. Pathol.*, **30,** 438.

RODE, W., SCANLON, K.J. & BERTINO, J.R. (1979). Thymidylate synthetase (EC 2.1.1.45) from L1210 mouse leukaemia cells—cell cycle pattern and affinity chromatography purification. *Dev. Biochem.*, **4,** 489.

ROSENBLATT, D.S. & ERBE, R.W. (1973). Reciprocal changes in the levels of functionally related folate enzymes during the culture cycle in human fibroblasts. *Biochem. Biophys. Res. Commun.*, **54,** 1627.

ROWE, P.B. (1971). The synthesis of $N^5$-$N^{10}$—methenyltetra-hydrofolic acid. *Meth. Enzym.*, **18B,** 733.

ROWE, P.B., TRIPP, E. & CRAIG, G.C. (1979). Folate metabolism in lectin activated human peripheral blood lymphocytes. *Dev. Biochem.*, **4,** 587.

SHIN, Y.S., WILLIAMS, M.A. & STOKSTAD, E.L.R. (1972). Identification of folic acid compounds in rat liver. *Biochem. Biophys. Res. Commun.*, **47,** 35.

SOTOBAYASHI, H., ROSEN, F. & NICHOL, C.A. (1966). Tetrahydrofolate cofactors in tissues sensitive and refractory to amethopterin. *Biochemistry*, **5**, 3878.

WHITEHEAD, V.M. (1971. Study of the folate polyglutamates in liver from animals and man. *Blood*, **38**, 809.

WHITEHEAD, V.M. (1973). Changes in rat liver folate composition and turn-over after partial hepatectomy. *Clin. Res.*, **21**, 571.

WITTWER, A.J. & WAGNER, C. (1980). Identification of folate binding protein of mitochondria as dimethylglycine dehydrogenase. *Proc. Natl Acad. Sci.*, **77**, 4484.